# Logicome Profiler: Exhaustive detection of statistically significant logic relationships from comparative omics data

**DOI:** 10.1371/journal.pone.0232106

**Published:** 2020-05-01

**Authors:** Tsukasa Fukunaga, Wataru Iwasaki

**Affiliations:** 1 Department of Computer Science, Graduate School of Information Science and Technology, The University of Tokyo, Tokyo, Japan; 2 Department of Biological Sciences, Graduate School of Science, The University of Tokyo, Tokyo, Japan; 3 Department of Computational Biology and Medical Science, Graduate School of Frontier Sciences, The University of Tokyo, Chiba, Japan; 4 Atmosphere and Ocean Research Institute, The University of Tokyo, Chiba, Japan; 5 Institute for Quantitative Biosciences, The University of Tokyo, Tokyo, Japan; 6 Collaborative Research Institute for Innovative Microbiology, The University of Tokyo, Chiba, Japan; University of Arizona, UNITED STATES

## Abstract

Logic relationship analysis is a data mining method that comprehensively detects item triplets that satisfy logic relationships from a binary matrix dataset, such as an ortholog table in comparative genomics. Thanks to recent technological advancements, many binary matrix datasets are now being produced in genomics, transcriptomics, epigenomics, metagenomics, and many other fields for comparative purposes. However, regardless of presumed interpretability and importance of logic relationships, existing data mining methods are not based on the framework of statistical hypothesis testing. That means, the type-1 and type-2 error rates are neither controlled nor estimated. Here, we developed Logicome Profiler, which exhaustively detects statistically significant triplet logic relationships from a binary matrix dataset (Logicome means *ome of logics*). To test all item triplets in a dataset while avoiding false positives, Logicome Profiler adjusts a significance level by the Bonferroni or Benjamini-Yekutieli method for the multiple testing correction. Its application to an ocean metagenomic dataset showed that Logicome Profiler can effectively detect statistically significant triplet logic relationships among environmental microbes and genes, which include those among urea transporter, urease, and photosynthesis-related genes. Beyond omics data analysis, Logicome Profiler is applicable to various binary matrix datasets in general for finding significant triplet logic relationships. The source code is available at https://github.com/fukunagatsu/LogicomeProfiler.

## Introduction

Recent technological advancements enabled us to obtain omics data from many samples for comparative purposes. In case of comparative genomics, it is now routine to obtain genome data from diverse species, and those data are frequently compared using a presence/absence binary matrix of encoded genes, or an ortholog table whose rows and columns mean species and genes. Such binary matrix representation is generally applicable to compare various types of omics data, such as genomics, transcriptomics, epigenomics, and metagenomics data across different samples. For example, metagenomic data from multiple sampling points can also be represented by a matrix whose rows and columns mean sampling points and genes [1]. Therefore, development of data mining methods for comparative omics matrices is a research topic of general importance in computational biology [2, 3].

Correlation analysis between two items is a typical method for analyzing comparative omics matrices. For instance, correlation analyses of gene expression and phylogenetic profiling matrices have estimated functions of function-unknown genes based on shared patterns of expression and occurrence between genes with related functions [4–6]. Even outside of the omics research fields, correlation analysis on ecology data matrices was also used to understand ecological interrelationships between species such as cooperation and competition [7]. However, biological items such as genes and species are often in complex relationships among more than two items, which cannot be revealed by such simple correlation analyses [8].

Logic relationship analysis is a method to discover relationships that fulfill logic-like conditions among three items in binary data matrices. For example, in the condition that C is present when A is not present but B is present and vice versa, the items A, B, and C (approximately) satisfy the logic condition C = ¬A ∧ B. While regression analysis with interaction terms can make a similar analysis for the logic condition C = A ∧ B, the logic relationship analysis can also detect various logic patterns such as C = (¬A) ∧ B as exemplified above. The Logic Analysis of Phylogenetic Profiles (LAPP) method was the first logic relationship analysis method, and its application to a phylogenetic profiling dataset succeeded to find triplet logic relationships about cell motility and intracellular traffic [9]. Since then, the LAPP method and its variants have been applied to various biological datasets such as gene co-expression and pathway data [10–12]. However, because the LAPP method detects logic relationships based on normalized mutual information, there is no guarantee that the detected logic relationships are statistically significant. In other words, the type-1 and type-2 error rates were neither controlled nor estimated in those studies.

In this study, we developed Logicome Profiler, which can exhaustively detect statistically significant logic relationships from binary matrices. To test all item triplets in a dataset while avoiding false positives, Logicome Profiler adjusts a significance level by the Bonferroni or Benjamini-Yekutieli method for the multiple testing correction. After examining the statistical property of the LAPP method, we applied Logicome Profiler to an ocean metagenomic dataset. The detected triplet logic relationships included those among urea transporter, urease, and photosynthesis-related genes in the marine microbial communities. Logicome Profiler was developed to effectively detect statistically significant triplet logic relationships from various comparative omics dataset, but is also applicable to any binary matrix dataset in general.

## Materials and methods

**Fig 1:**
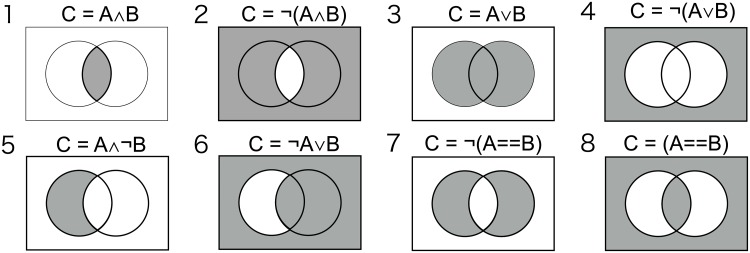
Venn diagrams and the logic formulas of 8 logic types.

Let *D* be a binary matrix dataset consisted of *N* samples and *K* items. *D*_*i*,*j*_, which is an element of the sample *i* and the item *j* on the matrix, takes either 0 or 1. Here, 0 and 1 mean absence and presence of the item in the sample, respectively. Logic relationship analysis considers 8 logic types, (1) C = A ∧ B, (2) *C* = ¬(*A* ∧ B), (3) C = A ∨ *B*, (4) *C* = ¬(*A* ∨ *B*), (5) C = A ∧ ¬*B*, (6) C = ¬A∨*B*, (7) *C* = ¬(*A* = = *B*), and (8) *C* = (*A* = = *B*) (Fig 1). We defined *l*_*i*_(*A*, *B*) as the *i*-th logic type between A and B (1 ≤ *i* ≤ 8). In addition, we defined |*A*| as the number of samples that A is present.

Intuitive interpretation of those eight logic types is presented below for reference by taking a comparative genomics case as an examples: (1) Both genes A and B are required for gene C; (2) Gene C can be replaced if both genes A and B are present; (3) Either gene A or B is required for gene C; (4) If gene A or B exists, gene C is not neccessary; (5) Gene C is required for gene A and gene B can replace gene A; (6) Gene C is not necessary if gene A exists but is necessary for gene B, and gene A is not necessary if gene B exists; (7) Gene C is required only either gene A or B exists; (8) Gene C exists only when both genes A and B exist or do not exist.

In addition, when an item is included in multiple detected logic relationships, it can be used for constructing a “logic network”. For example, Sprinzak *et al*. constructed a gene association network using gene triplets satisfying logic 1 and analyzed gene regulatory mechanisms for stress responses [11].

### The LAPP method

The LAPP method is the first data mining method for the logic relationship analysis [9]. This method first calculates three uncertainty coefficients *U*(*C*|*A*), *U*(*C*|*B*), and *U*(*C*|*l*_*i*_(*A*, *B*)) for all possible combinations of item triplets and logic types, where *U*(*X*|*Y*) = (*H*(*X*) + *H*(*Y*) − *H*(*X*, *Y*))/*H*(*X*). *H* denotes individual entropy or joint entropy in information theory. The numerator of *U*(*X*|*Y*) is mutual information, and thus the uncertainty coefficient means normalized mutual information in the range from 0 to 1. The large and small values mean that the value of *X* is predicted from the value of *Y* with high and low accuracy, respectively. Then, the LAPP method exhaustively detects triplet logic relationships that satisfy all of the following conditions (a) *U*(*C*|*l*_*i*_(*A*, *B*)) > 0.6, (b) *U*(*C*|*A*) < 0.3 and (c) *U*(*C*|*B*) < 0.3. That means, when the value of C is unpredictable from the value of the single item A or B but is predictable with high accuracy from the logic relationship between A and B, this method detects the triplet logic relationship. While the setting of the threshold values differs for each existing study, the LAPP method based on the uncertainty coefficients was the only method for the logic relationship analysis. Because we could not obtain the LAPP software implemented by the authors of the original article, we reimplemented this method and assessed its statistical property.

### Algorithm of Logicome Profiler

#### Hypothesis testing of logic relationships

**Fig 2:**
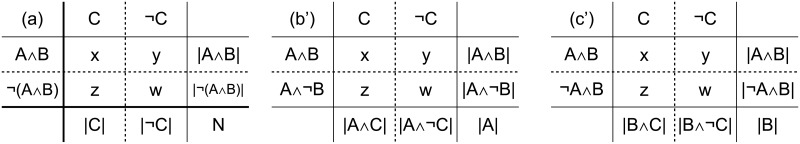
The contingency tables for the hypothesis tests of logic 1, C = A ∧ B.

Logicome Profiler detects logic relationships based on the framework of statistical hypothesis testing. We first describe the hypothesis testing method for a logic relationship using the logic 1, C = A ∧ B, as an example. Three conditions used in the LAPP method can be interpreted as follows: (a) *C* is associated with *A* ∧ B, (b) *C* is not associated with *A*, and (c) *C* is not associated with *B*. To make this approach statistically sound, we can straightforwardly conduct the hypothesis test of the condition (a) using the one-sided Fisher’s exact test (Fig 2). However, performing hypothesis tests of the conditions (b) and (c) is difficult for the following reason. In the framework of the Fisher’s exact test, the dependence of a variable to other variables can be statistically supported by rejecting null hypotheses of independence. On the other hand, the independence can NOT be statistically supported, because acceptance of null hypotheses of independence just means failure of rejection. Therefore, we re-wrote the conditions (b) and (c) as follows: (b’) *C* is associated with *A* ∧ B rather than *A* and (c’) *C* is associated with *A* ∧ B rather than *B*. In other words, Logicome Profiler investigates whether the logic relationship between two items A and B can explain the value of *C* more significantly than a single item A or B. These two conditions can be tested using the one-sided Fisher’s exact test like the condition (a) (Fig 2). The three conditions for all logic types used in Logicome Profiler are described in the S1 Text. We detected logic relationships whose all three tests are statistically significant. Note that we need not conduct multiple testing correction in this situation, because performing multiple hypothesis tests does not lead to the increase in false positives when all hypothesis tests must show statistical significances.

We can perform the hypothesis tests of logic types represented by conjunctive forms (the logics 1, 4 and 6) in the same way (S1 Fig). On the other hand, because logic types represented by disjunctive forms (the logics 2, 3 and 5) cannot be tested in this way, we changed the logic formulas to conjunctive forms by adding negation operators to both sides of the formulas. For example, C = A ∨ B, which is the logic 3, is changed to ¬C = ¬A ∧ ¬B. With this change, we can conduct the hypothesis tests for the logics 2, 3 and 5.

In the Venn diagram, areas corresponding to the logic types from 1 to 6 differ from that of a single term (A or B) or negation of a single term (¬A or ¬B) in only one area (Fig 1). Therefore, in the hypothesis tests of the conditions (b’) or (c’), the difference of the association to C between the single item and the logic relationship is clearly attributable to that area. On the other hand, the areas corresponding to the logic types 7 and 8 differ from that of a single term or negation of a single term in two areas. This means that it is difficult to identify the cause of the differences even if the hypothesis tests of the conditions (b’) or (c’) show statistical significances, and thus the interpretation of the results is difficult. Therefore, we excluded the logics 7 and 8 from the logic types analyzed by Logicome Profiler. The contingency tables for all logic types used in Logicome Profiler are described in S1 Fig.

#### Multiple testing correction

To comprehensively detect statistically significant triplet logic relationships, we have to conduct hypothesis tests for all candidate logic relationships. In that case, the number of hypothesis tests is *M* = 4*K*(*K* − 1)(*K* − 2). When mulple tests are performed simultaneously, the significance level has to be adjusted to avoid detection of many false positives. In this study, we adjusted the significance level using the Bonferroni method, which controls the family-wise error rate (FWER), and the Benjamini-Yekutieli method, which controls the false discovery rate (FDR), for multiple testing correction. FWER is the probability of finding one or more false positives when multiple tests are performed, and FDR is the expected ratio of false positives in all rejected hypotheses. The Bonferroni and Benjamini-Yekutieli methods obtain adjusted p-values by multiplying *M* and $\frac{M\log M}{k}$ by the original p-values, respectively. Here, *k* is the rank of the hypothesis when sorted by the p-values in the ascending order.

### Datasets for performance evaluation

To evaluate the performance of Logicome Profiler, we applied Logicome Profiler to simulated and empirical datasets. A simulated dataset contained 900 items that followed 300 logic relationships (50 for each logic type) that did not overlap each other. Then, for each logic relationship, presence/absence of two items were randomly determined with equal probabilities. Presence/absence of the third item was determined by strictly following the logic relationship at a 90% probability or randomly at a 10% probability. Each simulated dataset contained 100 samples, and 100 datasets were prepared.

As an empirical dataset, we used a metagenomic dataset provided by Tara Oceans Project [1]. This dataset includes metagenomic data from various water depth at 68 sampling points representative of worldwide oceanic regions. We used 105 samples from the water surface and the deep chlorophyll maximum layer. We used three matrices whose biological items are orthologous genes based on eggNOG database [13], orthologous genes based on KEGG OC database [14], and operational taxonomic units (OTUs). As the existence of each item for each sample is represented as a continuous value in the dataset provided by the original paper, we dichotomized these continuous values into binary values. We first normalized data so that the sum of abundance of items for each sample is 100.0, and set the threshold value to 5 × 10^−5^. Then, we removed the items that existed in over 85 or under 20 samples. This is because such items rarely appear in detected logic relationships because the information contents of these items are low. As a result, we obtained 797, 559, and 1580 items for the eggNOG ortholog dataset, the KEGG OC ortholog dataset, and the OTU dataset, respectively.

Note that the obtained dataset organization is highly sensitive to threshold values for the dichotomization. For example, when we changed the threshold value from 5 × 10^−5^ to 1 × 10^−5^ in the eggNOG dataset analysis, 797 and 1,821 OGs were identified, respectively, and only 48 OGs and 30 logic relationships (the significance level *α* was set to 0.001 for controlling FDR) were in common between them. We argue that such dependency on threshold values is a natural and proper consequence, because (1) different thresholds have different biological meanings (e.g., whether major or minor ecological functions are investigated) and (2) FWER and FDR are controlled in either case regardless of the threshold values.

## Results

### Assessment of statistical property of the LAPP method

**Fig 3:**
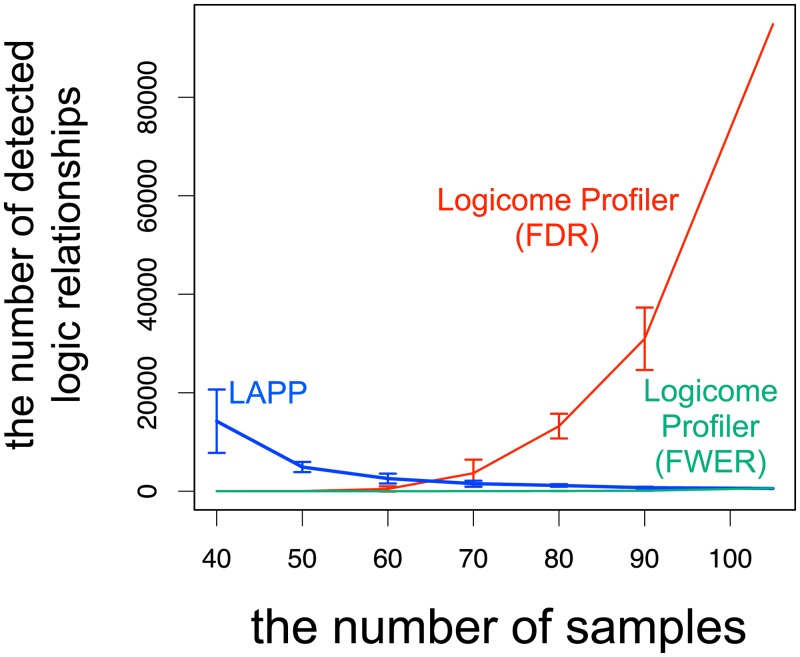
Dependence of the number of detected logic relationships on the number of samples. The x-axis and the y-axis represent the numbers of samples and detected logic relationships, respectively. The LAPP method, Logicome Profiler (FWER), and Logicome Profiler (FDR) are represented by blue, green and red lines, respectively. The error bars represent the standard deviations.

We first assessed statistical property of the LAPP method. The LAPP method cannot estimate and control the type-1 and type-2 error rates, and there is no guarantee that the LAPP method gives approximately constant error rates for any dataset size (number of samples). Accordingly, the effect of the number of sample to the number of detected logic relationships should be analyzed. We applied the LAPP method to the randomly sub-sampled eggNOG ortholog datasets. The numbers of samples of the sub-sampled datasets were from 40 to 90, and we created 10 sub-sampled datasets and calculated the average number of detected logic relationships for each number of samples. Fig 3 and S1 Table show how the number of detected logic relationships depends on the number of samples in the LAPP method. Unlike expected results in statistical analysis, the number of detected logic relationships in the LAPP method monotonically decreased with the increasing number of samples. This result means that the error rates of the LAPP method depend on dataset sizes, i.e., the LAPP method leads to large type-1 error rates when the dataset size is small but leads to large type-2 error rates when the dataset size is large. In real application, a user cannot know whether a dataset size gives large type-1 error rates or large type-2 error rates, and thus the dependence of error rates on the dataset size should make it difficult to interpret detection results. Therefore, a logic relationship analysis method based on the framework of statistical hypothesis testing, which can control the type-1 error rates, is definitely needed.

### Performance evaluation of Logicome Profiler

We evaluated the statistical power of Logicome Profiler by applying it to the simulated and empirical datasets. We first investigated whether Logicome Profiler can control FWER and FDR using simulated datasets. In this analysis, we set the significance level *α* to 0.05. When we controlled FWER based on the Bonferroni method, the empirical FWER was 0.03 and the average sensitivity was 0.30. On the other hand, when we controlled FDR based on the Benjamini-Yekutieli method, the empirical FDR was 0.04 and the average sensitivity was 0.90. These results show that Logicome Profiler can both fulfill the correction criteria for multiple testing correction and be effective for sensitive detection of significant logic relationships. We also examined numbers of detected logic relationships of Logicome Profiler using an empirical dataset. In this analysis, we set the significance level *α* to 0.05 and 0.001 for controlling FWER and FDR, respectively. Table 1 shows the numbers of detected logic relationships for both methods. It is notable that, in all datasets, Logicome Profiler controlling FDR detected many logic relationships although we set the significance level low. We finally examined the effect of the number of samples on the statistical power of Logicome Profiler. We used the same evaluation method as the evaluation of the LAPP method. Fig 3 and S1 Table show how the number of samples affects the numbers of detected logic relationships in Logicome Profiler. We verified that Logicome Profiler showed a statistically favorable property that the number of detected logic relationships monotonically increased with the increasing number of samples unlike the LAPP method.

**Table 1:** Numbers of detected logic relationships by Logicome Profiler.

Dataset	the Bonferroni method	the Benjamini-Yekutieli method
eggNOG	610	94855
KEGG OC	126	38555
OTU	6	25677

Then, we investigated basic properties of the results of Logicome Profiler. Hereafter, we focus on Logicome Profiler controlling FDR because Logicome Profiler controlling FWER detected only a few logic relationships. We first counted the number of detected logic relationships for each logic type (Table 2). In all datasets, the logic relationships of logics 1, 3, and 5 were frequently detected, whereas those of logics 2 were less detected. We secondly examined the occurrence frequency of each item in the detected logic relationships (S2 Fig). For all datasets, we found a tendency that a few items frequently appeared in the detected logic relationships. We thirdly checked the relationships between the occurrence numbers of items in the samples and those in the detection results (S3 Fig). As a result, there were no relationships in all datasets. This result means that whether an item is in the detected logic relationships cannot be predicted from the occurrence frequency of the item in the dataset.

**Table 2:** The number of the detected logic relationships for each logic type.

dataset	Logic 1	2	3	4	5	6
eggNOG	19202	135	26823	6360	37114	5221
KEGG OC	14833	36	5240	2351	13436	2659
OTU	4036	42	6497	2962	10217	1913

### Examples of the detected logic relationships

**Fig 4:**
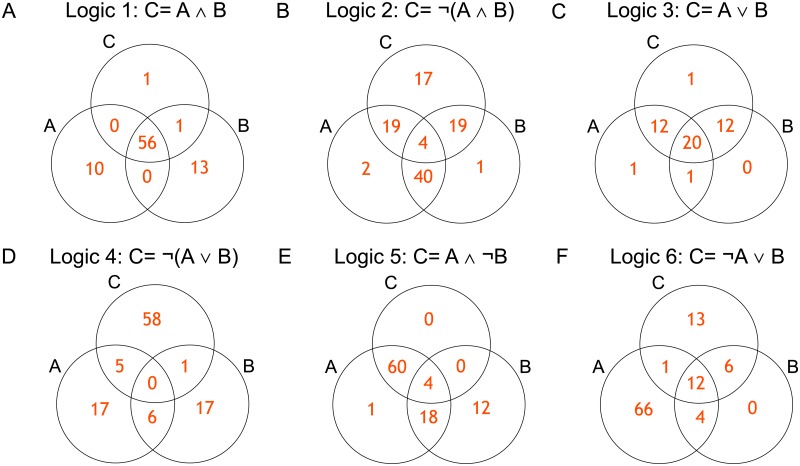
Visualization of examples of the detected logic relationships in the OTU dataset by Venn diagrams. (A) logic 1 (C = A ∧ B) (B) logic 2 (C = ¬(A ∧ B) (C) logic 3 (C = A ∨ B) (D) logic 4 (C = ¬(A ∨ B) (E) logic 5 (C = A ∧ ¬B) (F) logic 6 (C = ¬A ∨ B).

Here, we show some examples of the detected logic relationships to show the usefulness of Logicome Profiler. We first visualized an example of detected logic relationships in the OTU dataset for each logic type by the Venn diagram (Fig 4). The list of OTUs in these examples is in Table 3. For example, in most cases of Fig 4A and 4C was present only when both A and B were present, and C was absent when only either A or B was present. Therefore, this OTU triplet holded the logic 1 (C = A ∧ B). As another example, in most cases of Fig 4E and 4C was present only when A was present and B was absent, and thus this OTU triplet also holded the logic 5 (C = A #x2227; ¬B). These results suggest the existence of various statistically significant interspecific logic relationships.

**Table 3:** The list of OTUs in Fig 4.

	A	B	C
logic 1	Chloroplast HQ671986.1.1448	SAR324 EF573752.1.1474	Chloroplast EU268108.1.1451
logic 2	Myxococcales EF574964.1.1522	KI89A FJ744952.1.1345	Rhodospirillaceae HQ671902.1.1460
logic 3	Thaumarchaeota JF268338.1.1346	SAR11 HQ674219.1.1452	SAR11 EF646144.1.1435
logic 4	Acidimicrobiales HQ672763.1.1478	SAR11 FJ615107.1.1293	SAR116 EU795183.14183.15649
logic 5	SAR116 EF572060.1.1451	SAR202 AY534099.1.1476	OCS116 AACY023524552.82.1556
logic 6	SAR406 EF572866.1.1525	SAR86 EU800939.1.1503	SAR92 HQ672019.1.1489

Next, we investigated which genes tended to be enriched in the detected logic relationships. Table 4 and S2 Table are the lists of frequently occurred genes in the detected logic relationships of the KEGG OC ortholog dataset and the eggNOG ortholog dataset, respectively. In the list of the KEGG OC ortholog dataset, we found that urea transport system genes such as *urtA* were frequently observed. When we examined the detected logic relationships consisted of these genes, we discovered that these genes tended to show the following logic relationship: “urea transporter genes = urease genes ∧ component genes of photosystems”.

**Table 4:** The list of frequently occurred genes in the detected logic relationships in the KEGG OC ortholog dataset.

KEGG ID	gene name	frequency
K09121	*larC*	1834
K05808	*yhbH*	1468
K02699	*psaL*	1310
K11959	*urtA*	1231
K11963	*urtE*	1156
K06995	*unc*	1109
K06898	*larB*	1087
K11961	*urtC*	1047
K00273	*DAO*	988
K00320	*mer*	980

**Fig 5:**
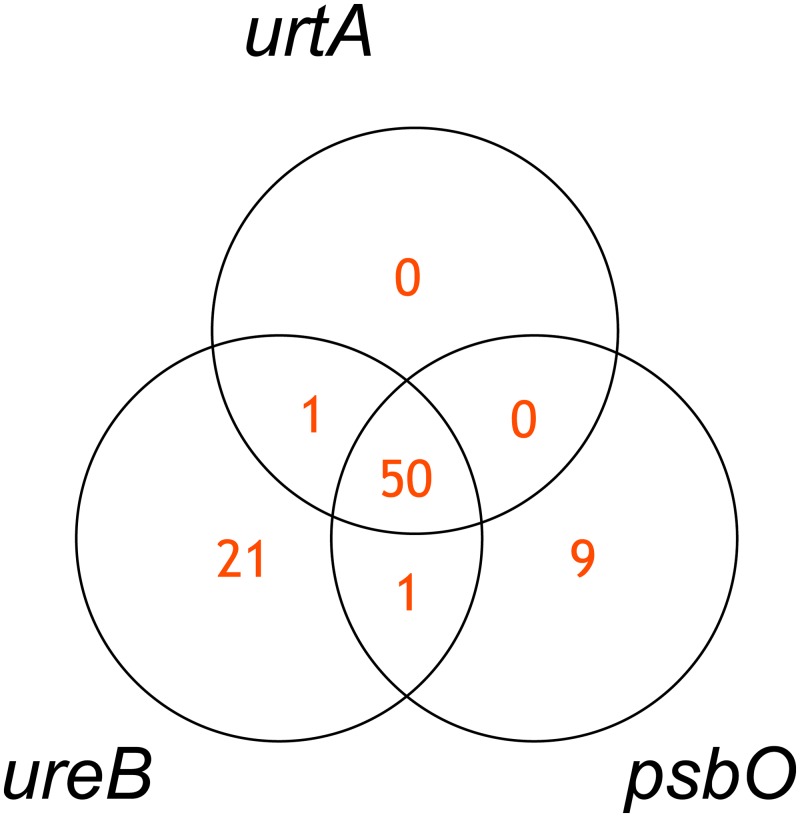
Visualization of “*urtA* = *ureB* ∧ *psbO*” by a Venn diagram.

Fig 5 shows the Venn diagram of “*urtA* = *ureB* ∧ *psbO*” as an example of the above-mentioned formula. In most cases, while *urtA* was present when both *ureB* and *psbO* were present, *urtA* was absent when only either *ureB* or *psbO* was present. The adjusted p-values of three hypotheses in this logic relationships were 2.4 × 10^−21^ (the condition (a)), 1.8 × 10^−11^ (the condition (b’)), and 2.4 × 10^−6^ (the condition (c’)), and thus logic 1 was certainly supported. Because nitrogen assimilation is involved in photosynthetic efficiency [15] and urea is one of the major nitrogen source in a group of cyanobacteria [16], the positive correlation relationships among *urtA*, *ureB* and *psbO* are generally reasonable. On the other hand, there are cyanobacteria species that utilize other nitrogen sources such as nitrate, cyanate and fixed nitrogen [15]. The presence of these species may lead to the presence of *psbO* in the absence of both *urtA* and *ureB*. In addition, a group of bacteria can take urea in cytoplasm by passive transportation depending on the concentration [17], and this may be the cause of the presence of *ureB* in the absence of both *urtA* and *psbO*. We assumed that these are rationales behind the detected logic relationships. In summary, Logicome Profiler can effectively identify biologically meaningful and important triplet logic relationships from comparative metagenomic data.

## Discussion

In this study, we proposed Logicome Profiler, which is a novel method for comprehensive detection of statistically significant triplet logic relationships.

We envision three future software extensions of Logicome Profiler. The first is to directly analyze data matrices consisting of continuous values. In this study, we dichotomized the continuous values into binary values based on a given threshold, but there would be two problems. The first is that giving a biologically meaningful threshold value would be difficult in many applications, and the second is that the association information among items may be lost due to the dichotomization. As a method of itemset mining analysis of continuous values, Tatti proposed a frequent pattern mining method based on an order statistics [18]. The application of the order statistics to the logic relationship analysis may also be a solution for detection of statistically significant logic relationships of continuous values.

The second is reducing false positives by removal of data biases derived from dependencies among samples. The Fisher’s exact test assumes independence of samples among a dataset, but the assumption often does not hold in biological data analysis. For example, there can be categorical covariates among samples or evolutionary relationships among species in phylogenetic profiling analysis. Since these dependencies among samples can lead to detection of many false positives, reduction of the data biases is important. In order to reduce the effect of categorical covariates, the application of the Cochran-Mantel-Haenszel test [19] or the exact logistic regression model [20] instead of the Fisher’s exact test would be effective. In addition, for the elimination of the influence of evolutionary relationships, some previous papers proposed usage of gain/loss of genes in branches of a reconstructed phylogenetic tree as items, instead of presence/absence of genes in species [21, 22]. The integration of these methods would increase the versatility of Logicome Profiler.

The third is the development of more sensitive detection approaches based on permutation testing methods. The Bonferroni method can control the FWER for multiple testing correction, but this method can not reach the optimal significance level for controlling FWER. Permutation testing methods such as the Westfall-Young method are alternative powerful approaches for controlling FWER [23]. These methods require a lot of computation time but have higher statistical power than the Bonferroni method, and thus several bioinformatics programs based on the permutation testing methods have been developed [24–26]. Logic relationship analysis based on the permutation-based testing methods would be useful especially when the number of samples of the dataset is small and a highly sensitive method is required.

## Conclusion

Logicome Profiler is the first method for the logic relationship analysis based on the framework of statistical hypothesis testing. For multiple testing correction, Logicome Profiler adjusts the significance level by the Bonferroni or Benjamini-Yekutieli method. We verified that Logicome Profiler effectively detects biologically meaningful triplet logic relationships using an ocean metagenomic dataset.

The knowledge discovery by Logicome Profiler is an important future perspective. Logic relationship analysis has been applied to genomic data, but Logicome Profiler is also applicable to any binary matrix data in general. Therefore, beyond omics data analysis or biological data analysis, the applications to protein domain data, chemical compound data, market data or natural language processing data are important research directions.

## Supporting information

S1 Text Functional enrichment analysis in the detected logic relationships. (PDF)

S1 Fig The contingency tables for the hypothesis tests. (A) Logic 1, C = A ∧ B. (B) Logic 2, C = ¬(A ∧ B). (C) Logic 3, C = A ∨ B. (D) Logic 4, C = ¬(A ∨ B). (E) Logic 5, C = A ∧ ¬B. (F) Logic 6, C = ¬A ∨ B.
(PDF)

S2 Fig Plots of the number of occurrence of the item in the detected logic relationships. (A) EggNOG ortholog dataset. (B) KEGG OC ortholog dataset. (C) OTU dataset.
(PDF)

S3 Fig Relationships between the number of occurrence of the item in the dataset and those in the detected logic relationships. (A) EggNOG ortholog dataset. (B) KEGG OC ortholog dataset. (C) OTU dataset.
(PDF)

S1 Table Dependence of the number of detections on the number of samples for each method. (PDF)

S2 Table The list of frequently occurred genes in the detected logic relationships in the eggNOG ortholog dataset. (PDF)
