# Supplementary material for: Logicome Profiler: Exhaustive detection of statistically significant logic relationships from comparative omics data
Source: PLoS One. 2020 May 1;15(5):e0232106. doi: 10.1371/journal.pone.0232106 (PMC7194410; doi:10.1371/journal.pone.0232106)
Supplement: S1 Text — (PDF) [file pone.0232106.s001.pdf]

Supplementary Text

Three conditiions for all logic types used in Logicome Profiler

logic 1

(a) C is associated with A $\wedge$ B

(b') C is associated with A $\wedge$ B rather than A

(c') C is associated with A $\wedge$ B rather than B

logic 2

(a) $\neg$C is associated with A $\wedge$ B

(b') $\neg$C is associated with A $\wedge$ B rather than A

(c') $\neg$C is associated with A $\wedge$ B rather than B

logic 3

(a) $\neg$C is associated with $\neg$A $\wedge$ $\neg$B

(b') $\neg$C is associated with $\neg$A $\wedge$ $\neg$B rather than $\neg$A

(c') $\neg$C is associated with $\neg$A $\wedge$ $\neg$B rather than $\neg$A

logic 4

(a) C is associated with $\neg$(A $\vee$ B) = $\neg$A $\wedge$ $\neg$B

(b') C is associated with $\neg$A $\wedge$ $\neg$B rather than $\neg$A

(c') C is associated with $\neg$A $\wedge$ $\neg$B rather than $\neg$B

logic 5

(a) C is associated with A $\wedge$ $\neg$B

(b') C is associated with A $\wedge$ $\neg$B rather than A

(c') C is associated with A $\wedge$ $\neg$B rather than $\neg$B

logic 6

(a) $\neg$C is associated with A $\wedge$ $\neg$B

(b') $\neg$C is associated with A $\wedge$ $\neg$B rather than A

(c') $\neg$C is associated with A $\wedge$ $\neg$B rather than $\neg$B
