# Supplementary material for: Logicome Profiler: Exhaustive detection of statistically significant logic relationships from comparative omics data
Source: PLoS One. 2020 May 1;15(5):e0232106. doi: 10.1371/journal.pone.0232106 (PMC7194410; doi:10.1371/journal.pone.0232106)
Supplement: S1 Fig — (A) Logic 1, C = A ∧ B. (B) Logic 2, C = ¬(A ∧ B). (C) Logic 3, C = A ∨ B. (D) Logic 4, C = ¬(A ∨ B). (E) Logic 5, C = A ∧ ¬B. (F) Logic 6, C = ¬A ∨ B. (PDF) [file pone.0232106.s002.pdf]

A.

(a)	C	$\neg C$	
$A \land B$	x	y	$\lvert A \land B \rvert$
$\neg(A \land B)$	z	w	$\lvert \neg(A \land B) \rvert$
	$\lvert C \rvert$	$\lvert \neg C \rvert$	N

(b’)	C	$\neg C$	
$A \land B$	x	y	$\lvert A \land B \rvert$
$A \land \neg B$	z	w	$\lvert A \land \neg B \rvert$
	$\lvert A \land C \rvert$	$\lvert A \land \neg C \rvert$	$\lvert A \rvert$

(c’)	C	$\neg C$	
$A \land B$	x	y	$\lvert A \land B \rvert$
$\neg A \land B$	z	w	$\lvert \neg A \land B \rvert$
	$\lvert B \land C \rvert$	$\lvert B \land \neg C \rvert$	$\lvert B \rvert$

B.

(a)	$\neg C$	C	
$A \land B$	x	y	$\lvert A \land B \rvert$
$\neg(A \land B)$	z	w	$\lvert \neg(A \land B) \rvert$
	$\lvert \neg C \rvert$	$\lvert C \rvert$	N

(b’)	$\neg C$	C	
$A \land B$	x	y	$\lvert A \land B \rvert$
$A \land \neg B$	z	w	$\lvert A \land \neg B \rvert$
	$\lvert A \land \neg C \rvert$	$\lvert A \land C \rvert$	$\lvert A \rvert$

(c’)	$\neg C$	C	
$A \land B$	x	y	$\lvert A \land B \rvert$
$\neg A \land B$	z	w	$\lvert \neg A \land B \rvert$
	$\lvert B \land \neg C \rvert$	$\lvert B \land C \rvert$	$\lvert B \rvert$

C.

(a)	$\neg C$	C	
$\neg A \land \neg B$	x	y	$\lvert \neg A \land \neg B \rvert$
$A \lor B$	z	w	$\lvert A \lor B \rvert$
	$\lvert \neg C \rvert$	$\lvert C \rvert$	N

(b’)	$\neg C$	C	
$\neg A \land \neg B$	x	y	$\lvert \neg A \land \neg B \rvert$
$\neg A \land B$	z	w	$\lvert \neg A \land B \rvert$
	$\lvert \neg A \land \neg C \rvert$	$\lvert \neg A \land C \rvert$	$\lvert \neg A \rvert$

(c’)	$\neg C$	C	
$\neg A \land \neg B$	x	y	$\lvert \neg A \land \neg B \rvert$
$A \land \neg B$	z	w	$\lvert A \land \neg B \rvert$
	$\lvert \neg B \land \neg C \rvert$	$\lvert \neg B \land C \rvert$	$\lvert \neg B \rvert$

D.

(a)	C	$\neg C$	
$\neg A \land \neg B$	x	y	$\lvert \neg A \land \neg B \rvert$
$A \lor B$	z	w	$\lvert A \lor B \rvert$
	$\lvert C \rvert$	$\lvert \neg C \rvert$	N

(b’)	C	$\neg C$	
$\neg A \land \neg B$	x	y	$\lvert \neg A \land \neg B \rvert$
$\neg A \land B$	z	w	$\lvert \neg A \land B \rvert$
	$\lvert \neg A \land C \rvert$	$\lvert \neg A \land \neg C \rvert$	$\lvert \neg A \rvert$

(c’)	C	$\neg C$	
$\neg A \land \neg B$	x	y	$\lvert \neg A \land \neg B \rvert$
$A \land \neg B$	z	w	$\lvert A \land \neg B \rvert$
	$\lvert \neg B \land C \rvert$	$\lvert \neg B \land \neg C \rvert$	$\lvert \neg B \rvert$

E.

(a)	C	$\neg C$	
$A \land \neg B$	x	y	$\lvert A \land \neg B \rvert$
$\neg(A \land \neg B)$	z	w	$\lvert \neg(A \land \neg B) \rvert$
	$\lvert C \rvert$	$\lvert \neg C \rvert$	N

(b’)	C	$\neg C$	
$A \land \neg B$	x	y	$\lvert A \land \neg B \rvert$
$A \land B$	z	w	$\lvert A \land B \rvert$
	$\lvert A \land C \rvert$	$\lvert A \land \neg C \rvert$	$\lvert A \rvert$

(c’)	C	$\neg C$	
$A \land \neg B$	x	y	$\lvert A \land \neg B \rvert$
$\neg A \land \neg B$	z	w	$\lvert \neg A \land \neg B \rvert$
	$\lvert \neg B \land C \rvert$	$\lvert \neg B \land \neg C \rvert$	$\lvert \neg B \rvert$

F.

(a)	$\neg C$	C	
$A \land \neg B$	x	y	$\lvert A \land \neg B \rvert$
$\neg(A \land \neg B)$	z	w	$\lvert \neg(A \land \neg B) \rvert$
	$\lvert C \rvert$	$\lvert \neg C \rvert$	N

(b’)	$\neg C$	C	
$A \land \neg B$	x	y	$\lvert A \land \neg B \rvert$
$A \land B$	z	w	$\lvert A \land B \rvert$
	$\lvert A \land \neg C \rvert$	$\lvert A \land C \rvert$	$\lvert A \rvert$

(c’)	$\neg C$	C	
$A \land \neg B$	x	y	$\lvert A \land \neg B \rvert$
$\neg A \land \neg B$	z	w	$\lvert \neg A \land \neg B \rvert$
	$\lvert \neg B \land \neg C \rvert$	$\lvert \neg B \land C \rvert$	$\lvert \neg B \rvert$

Fig. S1 The contingency tables for the hypothesis tests of (A) logic 1, $C = A \land B$, (B), logic 2, $C = \neg(A \land B)$, (C) logic 3, $C = A \lor B$, (D) logic 4, $C = \neg(A \lor B)$, (E) logic 5, $C = A \land \neg B$ and (F) logic 6, $C = \neg A \lor B$.
