# Supplementary material for: Logicome Profiler: Exhaustive detection of statistically significant logic relationships from comparative omics data
Source: PLoS One. 2020 May 1;15(5):e0232106. doi: 10.1371/journal.pone.0232106 (PMC7194410; doi:10.1371/journal.pone.0232106)
Supplement: S2 Fig — (A) EggNOG ortholog dataset. (B) KEGG OC ortholog dataset. (C) OTU dataset. (PDF) [file pone.0232106.s003.pdf]

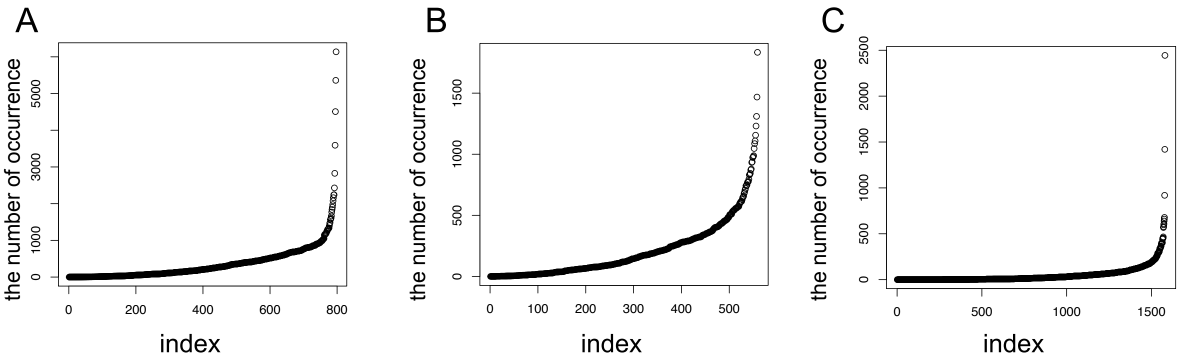


Fig. S2 Plots of the number of occurrence of the item in the detected logic relationships for (A) eggNOG ortholog dataset, (B) KEGG OC ortholog dataset and (C) OTU dataset. The x-axis and the y-axis represent the number of occurrence of the item and the index of the item, respectively. The items are sorted by the numbers of occurrence.
