# Supplementary material for: Logicome Profiler: Exhaustive detection of statistically significant logic relationships from comparative omics data
Source: PLoS One. 2020 May 1;15(5):e0232106. doi: 10.1371/journal.pone.0232106 (PMC7194410; doi:10.1371/journal.pone.0232106)
Supplement: S3 Fig — (A) EggNOG ortholog dataset. (B) KEGG OC ortholog dataset. (C) OTU dataset. (PDF) [file pone.0232106.s004.pdf]

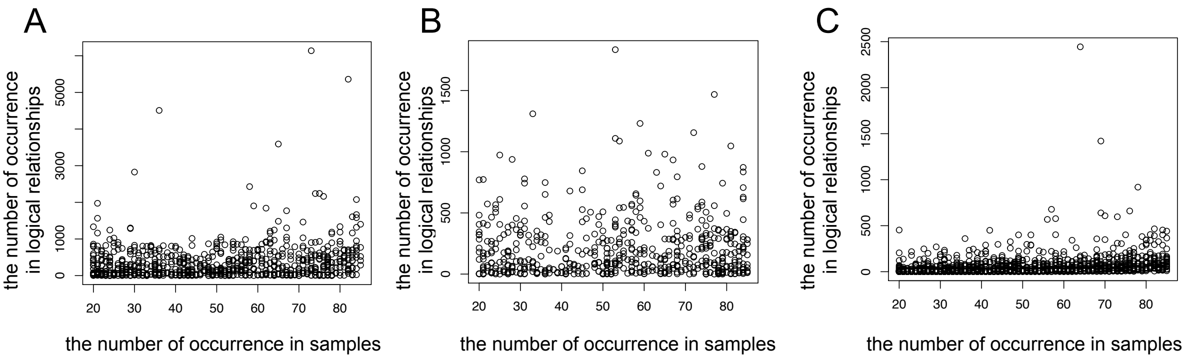

Fig. S3 Relationships between the number of occurrence of the item in the dataset and those in the detected logic relationships for (A) eggNOG ortholog dataset, (B) KEGG OC ortholog dataset and (C) OTU dataset. The x-axis and the y-axis represent the number of occurrence of the item in the dataset and that in the detected logic relationships, respectively.
