# Supplementary material for: Logicome Profiler: Exhaustive detection of statistically significant logic relationships from comparative omics data
Source: PLoS One. 2020 May 1;15(5):e0232106. doi: 10.1371/journal.pone.0232106 (PMC7194410; doi:10.1371/journal.pone.0232106)
Supplement: S1 Table — (PDF) [file pone.0232106.s005.pdf]

Table S1 Dependence of the number of detections on the number of samples for each method

	Method		
Sample size	LAPP	Bonferroni method	Benjamini-Yekutieli method
40	14214.3	0.0	0.0
50	4912.9	0.0	12.2
60	2560.7	0.1	456.3
70	1524.9	6.1	3655.3
80	1159.1	32.0	13215.6
90	707.2	91.0	30956.3
105	551	610	94855
