# Supplementary material for: Logicome Profiler: Exhaustive detection of statistically significant logic relationships from comparative omics data
Source: PLoS One. 2020 May 1;15(5):e0232106. doi: 10.1371/journal.pone.0232106 (PMC7194410; doi:10.1371/journal.pone.0232106)
Supplement: S2 Table — (PDF) [file pone.0232106.s006.pdf]

Table S2 The list of frequently occurred genes in the detected logic relationships in the eggNOG ortholog dataset

| COG/NOG ID | Description | Frequency |
|---|---|---|
| COG1661 | Predicted DNA-binding protein | 6137 |
| COG1836 | Predicted membrane protein | 5359 |
| COG2771 | DNA-binding HTH domain-containing proteins | 4508 |
| COG4333 | Uncharacterized protein | 3591 |
| NOG277419 | - | 2826 |
| NOG40089 | - | 2429 |
| COG0727 | Predicted Fe-S-cluster oxidoreductase | 2242 |
| NOG119063 | - | 2239 |
| COG3823 | Glutamine cyclotransferase | 2162 |
| COG1876 | D-alanyl-D-alanine carboxypeptidase | 2076 |

"-" means that the database had no annotation for the ortholog groups.
